# No evidence of associations between ADHD and event-related brain potentials from a continuous performance task in a population-based sample of adolescent twins

**DOI:** 10.1371/journal.pone.0223460

**Published:** 2019-10-04

**Authors:** Alex Lau-Zhu, Charlotte Tye, Frühling Rijsdijk, Grainne McLoughlin

**Affiliations:** 1 Social, Genetic and Developmental Psychiatry Centre, Institute of Psychiatry, Psychology and Neuroscience, King’s College London, London, England, United Kingdom; 2 Centre for Psychiatry, Brain Sciences Division, Imperial College London, London, England, United Kingdom; 3 Department of Child and Adolescent Psychiatry, Institute of Psychiatry, Psychology and Neuroscience, King’s College London, London, England, United Kingdom; University of Edinburgh, UNITED KINGDOM

## Abstract

We investigated key event-related brain potential markers (ERPs) derived from a flanked continuous performance task (CPT) and whether these would show phenotypic associations with ADHD (attention-deficit/hyperactivity disorder) in a population-based sample. We further explored whether there was preliminary evidence that such ERPs could also index genetic risk for ADHD (depending on finding phenotypic associations). Sixty-seven male-only twin pairs (*N* = 134; aged 12–15) from a subsample of the Twins’ Early Development Study, concordant and discordant for ADHD symptoms, performed the flanked CPT (or CPT-OX) while electroencephalography (EEG) was recorded. ERPs were obtained for cue (P3, CNV or contingency negative variation), go (P3, N2) and nogo trials (P3, N2). We found no phenotypic associations between CPT-derived ERPs and ADHD—the sizes of the estimated phenotypic correlations were nonsignificant and very small (*r*’s = -.11 to .04). Twin-model fitting analyses using structural equation modelling provided preliminary evidence that some of the ERPs were heritable (with the most robust effect for go-P3 latency), but there was limited evidence of any genetic associations between ERPs and ADHD, although with the caveat that our sample was small and hence had limited power. Overall, unlike in previous research, there was no evidence of phenotypic (nor preliminary evidence for genetic) associations between ADHD and CPT-derived ERPs in this study. Hence, it may be currently premature for genetic analyses of ADHD to be guided by CPT-derived ERP parameters (unlike alternative cognitive-neurophysiological approaches which may be more promising). Further research with better-powered, population-based, genetically-informative and cross-disorder samples are required, which could be facilitated by emerging mobile EEG technologies.

## Introduction

Attention deficit/hyperactivity disorder (ADHD) is characterised by persistent difficulties with attention, impulsivity and hyperactivity [1], with broad functional impact in domains such as education, relationships and employment [2,3]. Research in quantitative genetic (e.g., twin studies) has estimated up to 76% heritability for ADHD, with current consensus that multiple genes of small effects are likely to be implicated [4]. Critically, the mechanisms linking genes with behavioural manifestations of ADHD are yet fully understood, including potential pathways via neurocognitive systems [5–8]. Because the diagnosis of ADHD is currently conceptualised as the extreme end of a continuum of symptoms that are normally distributed in the population [9], studying ADHD dimensional traits can be complementary routes towards an improved understanding of the disorder.

A neurocognitive domain frequently studied in ADHD relates to attention and inhibition processes [5,8,10,11], which can be examined within a continuous performance task or test (CPT) [12,13]. In a typical CPT, participants monitor the appearance of an infrequent stimulus known as target, which could appear in the visual and/or auditory domain, in a sequence of distractor stimuli. CPT performance tends to be impaired in ADHD compared to control participants, indexed by increased omission errors (i.e., not responding to a target) and increased commission errors (i.e., responding to a distractor), as well as increased reaction time variability [12,14], though there are exceptions (e.g., [15]). Variants of CPT where the target is accompanied *with* flankers rather than alone have shown to be more sensitive to ADHD-control differences [16,17].

Recently, the clinical utility of CPTs for ADHD has been challenged despite their widespread use in clinical settings. A recent review found that, in children, the evidence for the use of CPTs for diagnosis of ADHD is inconsistent, and for medication management is limited, although some encouraging results suggest that CPTs may be more useful clinically when combined with measures of activity [18]. One study in adults with ADHD found that a CPT produced high false negative rates—only 51.7% of the adults were correctly classified as having ADHD [19]. In general, CPTs have been questioned for their lack of ecological validity, confound with IQ, as well as lack of discriminant validity to other disorders [20]. Even RT variability, once thought to be unique to ADHD, appears not be specific to it [21]. Nevertheless, CPTs remain a popular tool in research for studying attentional processes in ADHD (e.g., mainly though approaches that consider average group differences rather than individual classifications).

CPTs have been combined with neurophysiological techniques [8,13], including electrical event-related brain potentials (ERPs), derived from electroencephalography or EEG, which is an established neuroscience method in neurodevelopmental research [22,23]. ERPs index variety of sensory and cognitive processes as these occur in the order of milliseconds [24]. The most commonly assessed ERPs in CPTs are the P3 after the onset of cue, go and nogo trials; the N2 after the onset of go and nogo trials; and the contingency negative variation (CNV) which is a negative deflection after the onset of a cue trial and before the onset of the go trial [14,16,17,25–32].

ADHD has been phenotypically associated with reduced amplitudes of the cue-P3, nogo-P3, go-P3 and CN across children and adult samples. This pattern of results has been interpreted as reflecting impaired attentional orienting, response inhibition, response execution and response preparation, respectively [14,16,17,25,27,28,30–35]. ADHD tends *not* to be associated with reduced amplitude of the nogo-N2 (or go-N2) in the CPT—this paradigm is *not* thought to be sufficiently demanding to reliably elicit ADHD-control group differences in conflict monitoring [14,17,30]. It must be noted that several studies have failed to find the above positive associations [14,16,28–30,32,36,37] and such null findings are likely to be underestimated given the ‘file-drawer problem’ [38], even though it is strongly encouraged now to report null results [39]. One potential reason for the null is the heterogeneity of ADHD [40] and the nature of the samples, particularly as clinical samples can leave key variables uncounted for, such as disorder chronicity, medications and comorbid conditions. Some ADHD-related effects on ERPs indeed appear to be better explained by co-occurring externalizing behaviours [41,42], callous-unemotional traits [33] or autism spectrum conditions [14], rather than ADHD. These findings underscore the importance of population-based research to establish the reliability/generalisability of any potential ADHD-ERP associations.

Putative links between CPT-derived ERPs and ADHD point to candidate brain-based markers which could reflect pathways from genes to disorder, and if so CPT-derived ERPs may have the potential to guide genetic analysis of ADHD. Some recent studies suggest shared familial effects between ADHD and reduced nogo-P3 amplitude using a sibling design [43], or between ADHD and reduced nogo-P3/cue-P3 amplitudes using a family design [17]. Familial/sibling effects, however, cannot distinguish genetic from shared-environmental effects, unlike the classical twin design [44]. More fundamentally, a trait measure that is a candidate genetic-risk index must also be heritable in itself [45,46]. Data in adults (ages 18–28) point towards heritabilities of ~.50–60 for P3 and N2 amplitudes [47], with such magnitudes staying relatively stable across adulthood (ages 17–23) [48] and across early adolescence (ages 12–16) [49]. There is also evidence for the heritability of CNV in delayed response tasks (with estimates between ~.21 to ~.43, varying depending on task-load conditions and the channels considered; [50]). Nevertheless, these heritability estimates have been derived from heterogeneous paradigms, and heritability estimates on ERPs directly derived from CPTs are lacking. We should note that the search for consistent familial/genetic associations between ADHD and neurocognitive measures has so far only yielded inconsistent results (18).

In this study we investigated key event-related brain potential markers (ERPs) derived from a flanked continuous performance task (CPT), and whether these would show phenotypic associations with ADHD (attention-deficit/hyperactivity disorder) in a population-based sample of early adolescent twins. We further explored whether there was preliminary evidence that such ERPs could also index genetic risk for ADHD (depending on finding phenotypic associations). If so, we predicted the following conditions to be met [45,51]. First, ERPs (cue-P3, nogo-P3 and CNV amplitudes) derived from a flanked CPT (see Methods) would show phenotypic correlations with ADHD. Second, there would be some indication that such ERPs would be at least partly heritable. Third, if the above two conditions are met, then such ERPs would show preliminary evidence for genetic correlations with ADHD. With the current sample, we have previously found evidence of phenotypic and genetic associations between ADHD and other EEG-based oscillatory measures, including very low-frequency power (VLF; < .05 Hz) within the same flanked CPT as reported in the current paper [15], as well as between ADHD and theta-related activity during other rest/task conditions [52,53]. Here we present findings analysing key ERPs for the first time in this sample and using the flanked CPT.

## Method

### Sample

Participants were from the Neurophysiological Study of Activity and Attention in Twins (NEAAT), which has also been described elsewhere [15,52–54]. The NEAAT sample consisted of a subset of adolescent twin pairs from the Twins’ Early Development Study (TEDS), which is a longitudinal population-based study of all twins born in England and Wales in 1994 to 1996 [55]. The TEDS sample is highly representative of the general population in the United Kingdom, such as in terms of socioeconomic status, educational levels and ethnicity [55].

NEAAT participants were selected on a latent class trajectory analysis of ADHD symptom development over three timepoints (ages 8, 12 and 14), using a DSM-IV measure of ADHD symptoms [56]. The analysis was run using the COMPLEX option in the program MPLUS [57], with only male participants without medical conditions. This approach involved fitting a series of models including one to several more classes. We opted for three-class models which identified subgroups of individuals who have had stably high, middle or low symptoms of inattention and impulsivity/hyperactivity. Class membership proportions for low/middle/high inattention and impulsivity/hyperactivity scores were 18%/38%/44 and 20%/46%/33%, respectively.

The final NEAAT sample included participants with stably high (i.e., referred as ADHD in this paper) or stably low ADHD symptoms (i.e., non-ADHD control participants) of inattention and of impulsivity/hyperactivity across timepoints (ages 8, 12 and 14). Participants were as follow: 67 twin pairs in total, of which 22 pairs were concordant for high ADHD symptoms (monozygotic/MZ:11; dizygotic/DZ: 11); 8 pairs were discordant for ADHD symptoms (MZ:2; DZ: 6); 37 pairs were concordant for low ADHD symptoms (MZ: 21; DZ: 16). All participants were free of medication at the time of the study. The study was approved by King’s College London Psychiatry, Nursing and Midwifery Research Ethics Subcommittee (PNM/08/0-089). All participating parents gave written informed consent.

### Cued continuous performance task with flankers (flanked CPT)

The CPT-OX with flankers or flanked CPT [14,16,17,27,30,36] is a variant of the go/no-go task that probes for attentional orienting, response preparation, response execution and inhibition. There were four identical blocks of 100 trials each. Trials were presented in a pseudo-random sequence. On each trial, a black letter array was centrally presented, consisting of a centre letter flanked on each side by distractor letters. Each array was presented for 150 ms every 1650 ms. The centre letter was one of 11 letters subtending at 0.5 degrees (O, X, H, B, C, D, E, F, G, J, and L). On each trial, a centre letter was flanked by either ‘X’ or ‘O’ (except the centre letters ‘X’ and ‘O’ were always flanked by ‘O’ and ‘X’, respectively). Participants were instructed to respond to the central target letter ‘X’ (i.e., OXO) only when it was preceded by the central cue letter ‘O’ (i.e., XOX). There was a total of 40 cue-target sequences (i.e., XOX-OXO) and 40 cue-nontarget sequences (e.g., XOX-OHO). Viewing distance was kept consistent at 120 cm and the task duration was 11 min. Prior to the main task, participants underwent a short practice where task comprehension was ascertained verbally. The flanked CPT was preceded by a 6-min recording of EEG resting state [15] and followed by two other tasks not reported here [53]. Measures of CPT performance included omission errors (i.e., the number of targets missed), total commission errors (i.e., the number of responses to all nontargets), O-not-X commission errors (i.e., the number of responses to cue-nontarget arrays), mean reaction time (MRT; for correct target detection within 200 to 1500 ms post-target) and intra-subject variability in this RT (SDRT).

### Measures

#### Current ADHD symptoms

ADHD symptoms were assessed using the long version of the Parents Conner’s Rating Scale [56] on the day of testing, and the long version of the Teacher Conner’s Rating Scale [58] where available with phone-call follow-ups after completion of testing.

#### Cognitive ability (IQ)

Two web-based measures were collected as part of the ongoing TEDS study at age 14: the Wechsler Intelligence Scale for Children as a Process Instrument vocabulary multiple choice subtest as an index of verbal IQ [59] and Raven’s standard and advances progressive matrices as an index of non-verbal IQ [60]. Missing scores at age 14 were imputed from other available IQ scores at ages 7, 12 and 14. A g score was obtained by applying equal weights to both verbal and non-verbal IQ and adding up their standardised scores within the NEAAT sample. Measures of IQ and g are highly correlated and index general intelligence [61].

### ERP recording and processing

#### Recording

EEG was recorded using a 62-channel extended 10–20 system montage (BrainAmp DC; Brain Products, GmbH, Munich, Germany), with impedance kept below 5 kohm, and FCz as the recording reference electrode. Vertical and horizontal electrooculograms (EOGs) were recorded from electrodes placed above and below the left eye and at the outer canthi. The data were sampled at 500 Hz, stored and analysed offline.

#### Preprocessing

ERP analyses were performed using the ERPLAB package [62] within EEGLAB toolbox [63] for MATLAB (R2016a; [64]). The signal was digitally filtered at 0.1–30 Hz (−6 dB cut-off) and re-referenced to average reference. Channels with excessive noisy/technical problems were removed (based on extended periods of low correlation with neighbouring channels). Ocular artefacts were extracted using adaptive mixture independent component analysis (AMICA) [65,66]. Visual inspection was used to identify stereotyped components capturing ocular artefacts, which were then removed from the data by back-projecting only the remaining components to the channel data [67,68]. Segments with artefacts exceeding 200 μV in peak-to-peak in any channel were further rejected (based on [14]). Residual muscular artefacts were manually removed by visual inspection of the EEG (e.g., high-frequency and high-amplitude spikes) [24]. Missing channels were replaced with topographic spline interpolation so that the critical channels for the current scalp-based analyses (i.e., Cz, Pz and Fz) were available for all participants (in case these were removed as bad channels).

#### Processing

Stimulus-locked epochs were extracted (-200 to 1650 ms) and baseline-corrected (-200 to 0 ms). Epochs were averaged for *cue* trials (i.e., ‘XOX’), *go* trials which were correctly responded to (‘OXO’ preceded by ‘XOX’), and *no-go* trials which were correctly not responded to: any array but ‘OXO’ (i.e., a non-target) that was preceded by ‘XOX’ (i.e., a cue). Averages contained at least 19 trials (as in [14]; also see S1 Table). The selection of channels and latency windows for ERP analyses were based on previous studies using similar paradigms in ADHD research [14,16,17,27,30], based on where effects were expected to be maximal as well as visual inspection of the averaged ERPs and topographic maps for the current sample. For each ERP, we considered peak amplitude and peak latency measures; except for the CNV where we used area amplitude as in previous studies [14,16,17,27,30]: cue-P3 was measured at Pz (400–700 ms), go-P3 at Pz (200–500 ms), nogo-P3 at CZ (200–500 ms), go-N2 and nogo-N2 at Fz (200–400 ms), and CNV at Cz (1300–1650 ms). Note that when a single clear peak amplitude was not identified (e.g., go-P3), we used mean amplitude and 50% fractional-area latency initially (e.g., [14]). Such two alternatives to peak measures have shown to be less biased by noise [24]. But the pattern of results remained the same (i.e., no significant case-control differences), suggesting that noise is unlikely to account for our current null findings (see Results). Peak-based measures were reported for simplicity and consistency with previous studies, which reported ADHD-control differences with peak measures [14,16,17,27,30].

### Statistical analyses for baseline and performance data

Three participants were excluded for all analyses due to excessive artefact (*n* = 2; fewer than 19 artefact-free segments; as in our previous study [14]); or extreme commission errors that indicate low task engagement (*n* = 1). Group comparisons were performed using Stata [69]. To account for non-independent observations (i.e., twin pairs), baseline measures (age, IQ and current ADHD symptoms) were analysed using the regression command with robust clustering to estimate standard errors. As groups differed in age and IQ (Table 1), these effects were regressed out of performance scores and ERP measures, in line with our previous analyses using the same sample [15,52,53]. We also rerun the analyses without regressing out IQ, and the pattern of results remained broadly the same (S1 and S2 Tables). ADHD measures and performance scores were highly skewed and thus log-transformed using the lnskew0 command in Stata, before conducting group comparisons. Pearson-correlations were used to explore the association between age/IQ and each ERP measure. When relevant, Bayesian analyses with the Gönen’s method [70] were run in SPSS version 25 [71] to determine the relative likelihood under the null versus the alternative hypothesis for key ERPs most consistently shown to be linked to ADHD.

**Table 1:** Summary statistics and mean comparisons for age, IQ and ADHD measures adjusted for genetic-relatedness.

	ADHD (*n* = 52)	Control (*n* = 82)	*t*	*p*
**Age**	13.48 (0.78)	14.01 (0.92)	2.67	.01
**IQ** ^a^	97.33 (8.40)	103.82 (12.65)	2.92	.005
**Parents Conners Inattention subscale** ^b^^**,**^^d^	55.54 (8.83)	42.63 (3.06)	12.25	<.001
**Parents Conners Hyperactivity-Impulsivity subscale** ^b^^**,**^^d^	60.75 (12.24)	44.33 (2.25)	11.23	<.001
**Teachers Conners Inattention subscale** ^c^^**,**^^d^	56.50 (13.14)	49.16 (7.28)	2.08	.044
**Teachers Conners Hyperactivity-Impulsivity subscale** ^c^^**,**^^d^	57.54 (14.46)	51.31 (14.33)	1.49	.145

ADHD = Attention deficit/hyperactivity disorder.

^a^ IQ was estimated based on data at age 14 as part of web-based data collection for the Twins Early Development Study [55], primarily based on the Raven’s standard and advanced progressive matrices [60] and the WISC-III-PI multiple choice subtest [59], and with missing data imputed from multiple IQ subtests scores across ages 7, 12 and 14.

^b^ Long version of the Parent Conners’ Rating Scale T-scores [56] collected on the day of testing.

^c^ Long version of the Teacher Conners’ Rating Scale T-Scores [58] collected by contacting teachers after completion of the testing session.

^d^ Adjusted for age and IQ (see S1 Table for analyses without adjusting for IQ).

### Twin model-fitting on ERP data

#### Data preparation

The effects of age and IQ were also regressed out of the all ERP data due to associations with ADHD grouping (Table 1), before proceeding to twin model-fitting. This data preparation allowed for subsequent interpretation of the results specifically in terms of ADHD status (the aim of this study), above and beyond general cognitive ability or age-related typical neurodevelopmental changes. We also rerun the analyses without regressing out IQ, and the general pattern of results remained the same (S2–S5 Tables). Model-fitting were conducted using OpenMx package in R [72] with maximum likelihood statistics.

#### Twin correlations

A constrained correlational model was first fitted to the observed MZ and DZ data to estimate correlations between ADHD and key ERP measures (amplitude/latency of cue-P3, go-P3, nogo-P3, go-N2, nogo-N2, and CNV). The constrained model considered the same phenotypic correlation between each ERP measure and ADHD; 1 MZ and 1 DZ correlation for each ERP measure; and 1 MZ and DZ cross-trait cross-twin correlation between each ERP measure and ADHD. For each ERP, estimates were produced for i) the within-twin cross-trait correlation (correlation between ADHD and each ERP measure across zygosity), ii) the MZ and DZ cross-twin *within*-trait correlation (correlation between the same ERP measure between twins in the same pair), and iii) the MZ and DZ cross-twin cross-*trait* correlation (correlation between ADHD status in one twin and an ERP measure in the other twin in the same pair).

#### Genetic model-fitting

Twin model-fitting was performed using structural equation modelling (SEM) to model the differences in correlations between MZ and DZ twin pairs. A biometrical genetic model assumes that MZ twins and DZ twins share 100% and 50% of their genetic influences, respectively (but both pair types share 100% of their environmental influences). In a genetic bivariate liability-threshold model, the MZ:DZ ratio of the cross-twin within-trait correlations is used to decompose the variance of an ERP measure into additive genetic, common environmental and individual-specific environmental influences including measurement errors, referred to as A, C and E, respectively.

The MZ:DZ ratio of the cross-twin cross-trait correlations can be used to further decompose the potential covariation between ADHD and an ERP measure into genetic (rG), common environmental (rC) and individual-specific environmental (rE) correlations [73]. However, we did not proceed with the latter analyses, given the lack of any evidence that there was a genetic relationship between ERP measures and ADHD in this study (see Results for details), and thus we instead focused on exploring univariate analyses of the ERP measures.

#### Ascertainment correction

The heritability of ADHD cannot be estimated due to the selected nature of our sample. Because selection is through ADHD traits but blind to ERP values, the necessary correction would depend only on the model for ADHD. Hence to obviate this correction one could alternatively fix the model parameters for ADHD (prevalence and variance components) to constant values. Thus, ADHD status (yes = high ADHD symptoms; no = low ADHD symptoms) was modelled using liability threshold, fixed to a population prevalence for ADHD at around 5% [74]. This model assumes that ADHD risk is normally distributed on a continuum and ADHD occurs when the threshold is exceeded [75]. MZ and DZ cross-twin correlations on ADHD status were fixed based on heritability estimates from a meta-analysis [4], with the following parameters: *h*^2^ = .76, *c*^2^ = .00, *e*^2^ = .24, consistent with rMZ = .76 (*h*^2^+*c*^2^) and rDZ = .38 (.5*h*^2^+*c*^2^). We have established, validated and successfully applied this model to investigate the link between EEG [15,52,53] and cortisol outcomes [54] with ADHD, as well as the link between brain-based measures and other low-prevalence conditions, such as schizophrenia [76–79], bipolar disorder [80,81], and psychopathy [82].

## Results

### Group comparisons

First, analyses on group comparisons are presented between ADHD and control participants (i.e., high versus low ADHD symptoms), who differed in both age and IQ (Table 1). As already reported previously on this sample [15], there were no significant group differences in any CPT performance measure when controlling for age and IQ (Table 2; see S3 Table for analyses without controlling for IQ).

**Table 2:** Summary statistics and mean comparisons for performance scores and ERPs on the flanked CPT controlling for age, IQ and genetic-relatedness.

		ADHD	Control	*t*	*p*
Performance		(*n* = 52)	(*n* = 82)		
**Omissions**		1.94 (2.35)	0.91 (1.60)	1.51	.135
**Commissions**		2.56 (3.14)	2.10 (2.43)	0.38	.707
**MRT (ms)**		422.48 (66.24)	396.45 (56.42)	1.46	.149
**SDRT (ms)**		112.75 (53.03)	86.84 (38.14)	1.90	.062
**CV**		0.26 (0.11)	0.21 (0.08)	1.81	.075
**ERPs**^a^		**(*n* = 50)**	**(*n* = 81)**		
**Cue-P3**	**Peak amplitude (μV)**	11.93 (4.78)	11.58 (4.96)	0.14	.886
	**Peak latency (ms)**	545.49 (79.35)	524.47 (72.90)	0.79	.430
**Go-P3**	**Peak amplitude (μV)**	16.48 (4.38)	17.09 (6.24)	1.05	.298
	**Peak latency (ms)**	422.85 (105.30)	411.65 (97.89)	0.17	.869
**NoGo-P3**	**Peak amplitude (μV)**	13.40 (5.83)	14.30 (5.74)	0.39	.697
	**Peak latency (ms)**	432.43 (63.76)	416.22 (67.07)	0.11	.915
**Go-N2**	**Peak amplitude (μV)**	-5.39 (3.27)	-4.87 (4.25)	0.22	.824
	**Peak latency (ms)**	312.45 (41.04)	304.29 (41.92)	0.66	.509
**NoGo-N2**	**Peak amplitude (μV)**	-7.14 (3.97)	-6.94 (4.49)	0.08	.936
	**Peak latency (ms)**	297.67 (35.94)	294.80 (31.61)	0.12	.908
**CNV**	**Area amplitude (μV)**	1.81 (1.06)	1.71 (0.89)	0.62	.537

CPT = continuous performance task; ADHD = attention deficit/hyperactivity disorder; MRT = mean reaction time; SDRT = standard deviation of reaction time; CV = coefficient of variation (SDRT/MRT); ERP = event-related potential

^a^ ERPs were obtained from fewer participants than performance scores due to data loss in EEG pre-processing.

**Fig 1:**
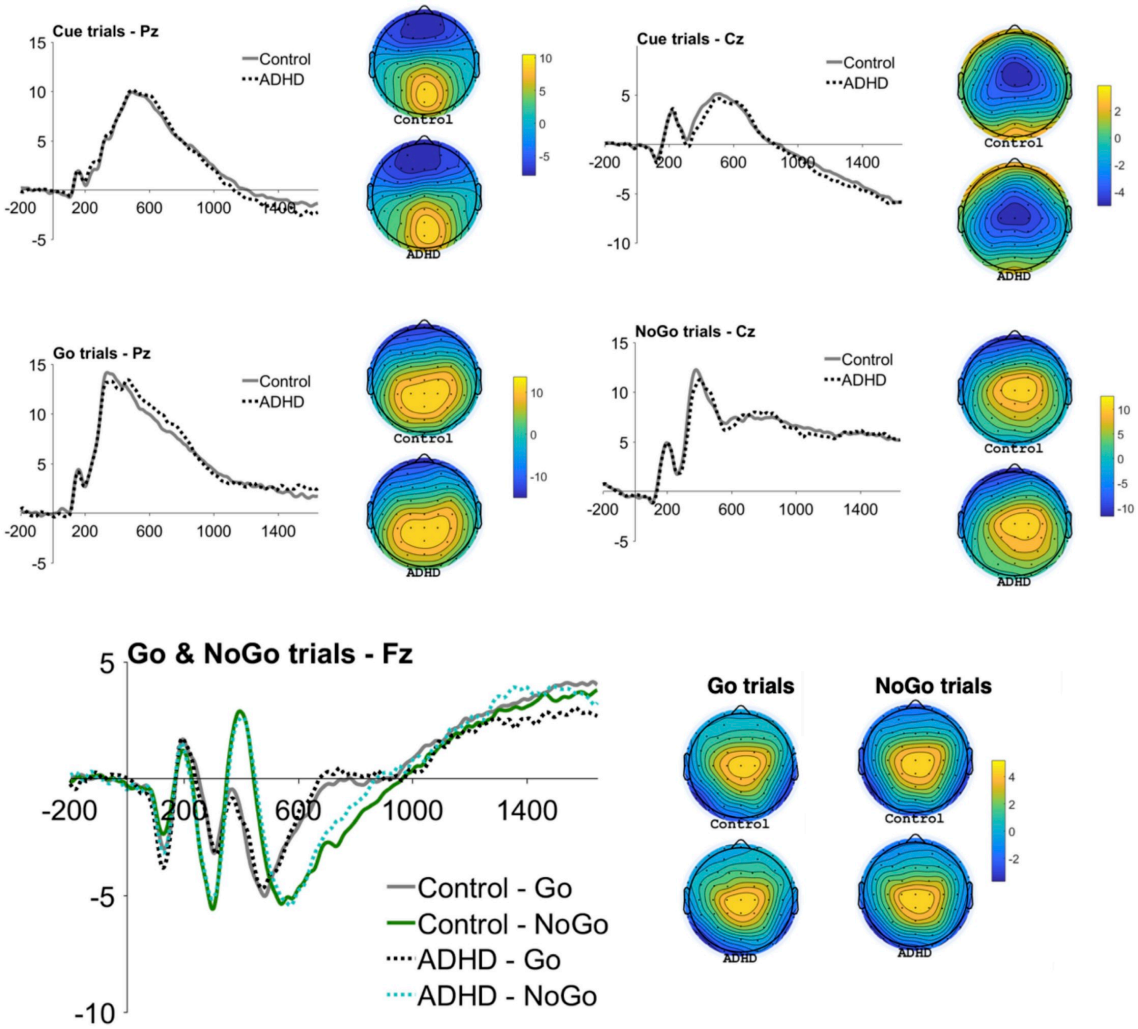
Grand mean event-related potentials (ERPs) to cue stimuli at Pz (for P3) and Cz (for CNV), to go stimuli at Pz (for P3) and Fz (for N2), and to nogo stimuli at Cz (for P3) and Fz (for N2), and the corresponding topographic maps by group.

ERPs from the flanked CPT were identified, namely cue-P3, go-P3, nogo-P3, CNV, go-N2 and nogo-N2 (Fig 1). In line with previous studies, nogo trials elicited higher N2 amplitudes (negative deflection) than go trials, *t*(130) = 5.74, *p* < .001 (Fig 1), but there were no significant group differences in any ERP measure, for either amplitude or latency (Table 2), even when *not* controlling for IQ (S3 Table). We run Bayesian analyses using a conservative medium effect size as the prior (*d* = .50) based on reported medium-to-large effect sizes from previous literature [14,17,35]. The associated Bayes factors for cue-P3, nogo-P3 and CNV amplitudes were 5.67, 4.75 and 5.17, all of which were above 3 and hence can be interpreted as evidence for the null [83,84].

In additional exploratory analyses, we found that across the whole sample regardless of ADHD grouping, IQ was positively correlated with nogo-P3 amplitude (*r* = .20, *p* = .027) and negatively correlated with nogo-P3 latency (*r* = -.22, *p* = .014), whereas age was negatively correlated with cue-P3 amplitude, cue-P3 latency and nogo-P3 latency (*r*’s = -.23 to -.18, *p*’s < .036).

### Twin model-fitting on ERP data

The maximum likelihood correlations of each ERP measure and ADHD were derived from the full constrained correlational model (Table 3). The same pattern of results was found even if IQ scores were not regressed out prior to twin model-fitting (S4 and S5 Tables).

**Table 3:** MZ and DZ cross-twin within-trait correlations for all ERP measures and cross-twin cross-trait correlations between ADHD and all ERP measures from the flanked CPT.

		Estimate (95% CI)^a^				
		Phenotypic Correlation with ADHD	Cross-Twin Within-Trait Correlation		Cross-Twin Cross-Trait Correlation with ADHD	
			MZ	DZ	MZ	DZ
**Cue-P3**	**Amplitude**	-.05 [-.26 to .16]	.45 [.17 to .65]*	-.05 [-.42 to .34]	-.04 [-.33 to .25]	.09 [-.16 to 33]
	**Latency**	.03 [-.15 to .22]	.12 [-.20 to .41]	.33 [-.10 to .61]	.29 [-.02 to .50]	-.03 [-.27 to .21]
**Go-P3**	**Amplitude**	-.01 [-.34 to .20]	.11 [-.21 to .40]	.20 [-.17 to .51]	-.31 [-.53 to .30]	.05 [-.21 to .31]
	**Latency**	.-02 [-.21 to .17]	.48 [.17 to .69]*	-.11 [-.46 to .28]	.16 [-.09 to .39]	.16 [-.08 to .38]
**NoGo-P3**	**Amplitude**	-.11 [-.30 to .10]	.35 [-.06 to .61]	-.02 [-.33 to .28]	.14 [.-.21 to .42]	.10 [-.14 to .33]
	**Latency**	.03 [-.18 to .23]	.21 [-.13 to .49]	-.16 [-.48 to .22]	-.03 [-.31 to .27]	-.06 [-.29 to .17]
**Go-N2**	**Amplitude**	-.00 [-.20 to .21]	.34 [.00 to .59]	-.21 [-.52 to .16]	.12 [-.15 to .35]	.02 [-.24 to .27]
	**Latency**	.04 [-.16 to .23]	.08 [-.24 to .37]	.06 [-.42 to .49]	-.22 [-.43 to .05]	.07 [-.19 to .33]
**NoGo-N2**	**Amplitude**	.02 [-.18 to .22]	.44 [.17 to .64]*	.22 [-.22 to .55]	-.07 [-.32 to .17]	.04 [-.22 to .29]
	**Latency**	.04 [-.18 to .26]	.05 [-.45 to .49]	-.17 [-.42 to .10]	.06 [-.29 to .38]	-.17 [-.38 to .07]
**CNV**	**Amplitude**	.01 [-.19 to .20]	.37 [.04 to .61]*	.09 [-.25 to .41]	.01 [-.24 to .27]	.12 [-.12 to .34]

MZ = monozygotic; DZ = dizygotic; ERP = event-related potential; ADHD = attention deficit/hyperactivity disorder; CPT = continuous performance task; CI = confidence intervals; CNV = continency negative variation

^a^ The MZ and DZ correlations for ADHD were fixed to population values to account for the selected sample with rMZ = .76, rDZ = .38 and a threshold for population prevalence of 5%; all ERP outcomes were peak measures, except for CNV which was area amplitude.

*p < .05

#### Phenotypic correlations

We also found no reliable phenotypic associations between any ERP measures and ADHD in this analysis—all effects were very small and nonsignificant (Table 3)—in line with the analyses using group comparisons reported above (Table 2). Note that unlike the linear regression approach, this twin-model fitting approach considered an ascertainment correction (due to the selected nature of our sample) and hence provided a more precise estimate of the phenotypic associations between ADHD and ERP parameters.

#### Cross-twin within-trait correlations (of ERPs)

We found, first, a significant MZ correlation, alongside a lower DZ correlation, was present for cue-P3 amplitude, go-P3 latency, nogo-N2 amplitude and CNV, which is evidence for genetic influences. Second, all MZ correlations deviated from one, suggesting the presence of non-shared environmental effects. Third, none of the DZ correlations were significant, suggesting that there was no evidence for shared environmental influences of any of the ERP measures. Fourth, the MZ correlation was more than half of the DZ correlation for the above ERP measures, which suggest nonadditive genetic dominance effects. However, only broad-sense heritable effects (additive and non-additive) were considered here due to the lack of power in our relatively small sample [85], given the constraints of combining twin design with lab-based brain measures.

#### Cross-twin cross-trait correlations

We found none of the relevant MZ correlations were significant (Table 3), which suggest that none of the ERP measures and ADHD share genetic influences. Given the lack of phenotypic correlations between ERP measures and ADHD (as shown by both analyses using group comparisons with linear regressions as well as constrained correlational model), and the absence of any significant MZ cross-twin *cross*-trait correlations, further analyses to estimate genetic correlations were not pursued.

#### Heritability estimates of ERPs

Exploratory analyses from now are focused on estimating the heritability of those ERP measures with significant MZ cross-twin *within*-trait correlations (cue-P3 amplitude, go-P3 latency, nogo-N2 amplitude and CNV), using univariate models for each ERP measure (Table 4). Note that the cross-twin within-trait correlation for the nogo-P3 amplitude failed to reach significance. However, its heritability was still estimated for the following reasons: a significant estimate for familiality has been previously reported [43]; its MZ within-trait correlation in the current study had a small-to-moderate size (*r* > .20) and was significant when IQ was not regressed-out (S5 Table).

**Table 4:** Standardized estimates of genetic, shared and nonshared environmental contributions to the variance of ERPs (from the flanked CPT) in univariate twin analyses.

	Estimate (95% CI)^a^		
	*h*^*2*^	*c*^*2*^	*e*^*2*^
**Cue-P3 amplitude**	.40 [.00 to .62]	0 [.00 to .38]	.60 [.38 to .89]*
**Go-P3 latency**	.45 [.004 to .67]*	0 [.00 to .30]	.55 [.33 to .86]*
**NoGo-P3 amplitude**	.28 [.00 to .57]	0 [.00 to .32]	.72 [.43 to 1]*
**NoGo-N2 amplitude**	.44 [.00 to .63]	0 [.00 to .53]	.56 [.36 to .83]*
**CNV amplitude**	.35 [.00 to .59]	0 [.00 to .42]	.65 [.41 to .96]*

ERP = event-related potential; CPT = continuous performance task; CI = confidence intervals; *h*^2^ = addictive genetic influences; *c*^*2*^ = shared environmental influences; *e*^*2*^ = nonshared environmental influences and measurement error

^a^ For parsimony and simplicity, we focused on univariate models for selected ERP measures because 1) these showed significant MZ cross-twin within-trait correlation, 2) there were no significant phenotypic associations ADHD grouping and any of the ERP measures, and 3) there were no significant cross-twin cross-trait correlations between any ERP measures and ADHD grouping.

*p < .05.

Results from SEM suggested that genetic factors may have small to moderate effects on individual differences in key ERPs derived from the flanked CPT. However, potentially due to the lack of power in our small twin sample, only the heritability estimate of go-P3 latency did not overlap with zero, suggesting that this estimate is the only robust one given our sample size. Shared environment did not appear to contribute to such individual differences, and nonshared environment (alongside measurement error) appeared to have at least moderate effects.

## Discussion

This study used a population-based twin sample in early adolescence to test for phenotypic (and potentially genetic) associations between ERP parameters (derived from a flanked CPT) and ADHD. Critically, we found that these ERPs were not phenotypically associated with ADHD—the size of the estimated phenotypic correlations between ADHD and ERPs were nonsignificant and also very small (*r*’s = -.11 to .04). We also found preliminary evidence that these ERPs may be heritable (with the strongest evidence for go-P3 latency), although caution is required as most confidence intervals overlapped with zero due to our small sample. There appear to be no sufficient evidence for genetic correlations between ADHD and ERPs—this may again be due to low power (but the lack of power to detect cross-twin cross-trait correlations is not of particular theoretical interest here given the non-significant and very small estimates for the phenotypic associations). With phenotypic correlations of around .02 (estimated in this sample) we would need thousands of twin pairs to detect a significant effect—such a small association, even if significant, would not be of theoretical interest or useful for genetic analyses [40,86].

The absence of ADHD-related effects on cue-P3, nogo-P3 and CNV amplitudes may seem surprising, given previous positive findings in other samples [14,17,25,28,31,32]. However, a closer look into the literature revealed that similar null findings have also been reported albeit to a lesser extent, including for cue-P3 [29,30], nogo-P3 [32], go-P3 [16,28,37], and CNV [14,36]. Some of these null findings have been reported in dissertations and not yet formally published in peer-reviewed journals [e.g., 36]. The lack of phenotypic associations between the N2 ERPs and ADHD is consistent with previous studies, as these effects are thought to be less salient unless the tasks used are more attentionally-demanding, unlike the flanked CPT [14,16,17,30]. We note that others have also recently questioned the utility of CPTs for identifying individuals with ADHD [18,19], and our data appear to be consistent with research failing to find phenotypic associations between CPT-based outcomes and ADHD symptoms [18–20].

Some may argue that our null phenotypic findings were due to the use of peak-based ERPs which could be susceptible to noise. We argue against such a possibility: previous positive findings were also mostly based on peak-based measures; the null in the present study found evidence with a Bayesian approach; the null remained after considering nonpeak-based alternatives (e.g., mean amplitudes) known to be less susceptible to noise (see Methods); our analytical approach, including the pre-selection of channels, followed closely previous research using the same flanked CPT paradigm, thus avoided analytical flexibility that has long hampered the ERP literature (see Methods); and finally—despite the lack of significant ADHD-control differences—we were able to find across the whole sample significant ERP differences between go and no-go trials as well as correlations between ERPs and age/IQ, indicating our EEG data was successfully preprocessed to allow for such signal to emerge from any ‘noise’.

As positive findings predominate the literature, we have yet to learn the true extent of similar null results. We believe that it is important to publish our null results even if somehow ‘unexpected’. This evidence base (e.g., to inform formal meta-analyses) is critical for establishing any reliable finding in science, including reliable biomarkers in psychiatry [39]. Given the heterogeneity of ADHD [40], moderators of these effects may remain unexplored if only positive findings were reported. Some studies have suggested that ADHD-related effects on ERPs may be better explained by co-occurring conditions co-occurring externalizing behaviours [41,42], callous-unemotional traits [33] or autism spectrum conditions [8,14]. One could argue that many of these confounds are likely to exist in research designs with clinical samples (which most of the positive findings rely on), including disorder chronicity, medication use and comorbidities, and therefore with more severe impairments than our sample. A strength of our study is the use of a population-based (non-clinical) cohort, with ADHD grouping derived from longitudinal trajectories of symptom development. However, as we did not consider diagnoses here, we were unable to directly compare those individuals with ADHD and a formal clinical diagnosis for the disorder versus those with ADHD but without such a diagnosis. Nevertheless, it is conceivable that findings derived from clinical samples may not always generalize to non-clinical samples. Future research could combine population-based ascertainment with diagnostic procedures, and include more comprehensive assessments of co-occurring features (e.g., callous-unemotional traits, autism, and mental health problems). The need for large-scale EEG-based studies is increasingly recognised in neurodevelopmental research [8,22] and we note some are underway (https://gtr.ukri.org/projects?ref=MR%2FN013182%2F1).

An important limitation of our study is low power due to the small sample size for a twin study. Based on our simulations, we have estimated that the sample size needed to detect a *h*^*2*^ of an ERP measure of .40 (with 80% power, and the *h*^*2*^ of ADHD fixed to .80 and the prevalence to 5%) is between 48–72 twin pairs, and to detect a *rg* of .40 is between 102–206 twin pairs (the specific numbers depend on the configuration of available twin pairs based on different combinations of concordance, zygosity and affected status). The phenotypic correlations associated with a *rg* of .40, and *h*^*2*^ ERP = .40 would be .26, which is way above what we observe in this sample. With this caveat in mind, we first estimated the heritability of key ERPs derived from the flanked CPT in an early adolescence, as previous studies focused mainly on late adolescence [49] and adulthood [47,48]. We found significant MZ cross-twin within-trait correlations for all ERPs (Table 3) which support the presence of genetic influences. The corresponding heritability estimates of these ERPs appeared to be mostly moderate, between .28 and .45 (Table 4). However, the confidence intervals overlapped with zero (except for go-P3 latency) hence larger samples are needed to confirm these estimates. We also intended to estimate the genetic correlations between ERPs and ADHD but found no significant MZ cross-twin cross-*trait* correlations (Table 3), which could indicate lack of genetic associations but also had lack of power. Nevertheless, the lack of power to detect potential cross-twin cross-trait correlations between ADHD and ERPs is not of particular interest given the lack of phenotypic associations.

Previous findings using this same subsample of TEDS (i.e., NEAAT) could help provide a wider context in which to interpret our current null results. Using the same twin sample and the same flanked CPT, we previously found significant phenotypic and genetic correlations between ADHD and very-low frequency power (< .05 Hz) [15]. We also found significant phenotypic and genetic correlations between ADHD and theta power during rest [52] and to trial-by-trial theta phase-variability within another attentionally-demanding flankers task [53]. A possibility is that oscillatory approaches (which the positive findings in the NEAAT sample rely on) represent more sensitive tools of genetic analyses. Traditional ERP analyses—as in our current study but also others [79,81,87,88]–include only time-locked and phase-locked EEG activity in relation to an event but with the majority of ‘background’ EEG filtered out. Such an approach may ignore the full extent of the brain dynamics [89] because ongoing oscillatory activities (even those not time- or phase-locked) are theorised to play critical roles in cognition, by coding information within and across neural circuitry and modulating neural excitability [90–93], hence possibly impacting mental health [94,95]. Future research could also capitalise on advanced computational approaches, such as the use of ICA, to ‘unmix’ channel-based signals into their constituent sources [66,89,96]–an approach which is potentially more informative for genetic and psychopathology research [23,53,97–99].

## Conclusions

In a population-based sample of adolescent twins (a small subsample of TEDS; *N* = 67), key CPT-derived ERPs were unexpectedly not associated with ADHD, despite previous findings indicating that such associations would have been found. Hence it may be currently premature for genetic analyses of ADHD to be guided by CPT-derived ERP measures (unlike alternative neurophysiological indices such as those using oscillatory-based approaches). Taken together, our data highlight the importance of large-scale, population-based, genetically-informative and cross-disorder designs [8] to investigate reliable genetic risk markers/pathways in neurodevelopmental disorders using functional brain measures. Such an endeavour could be propelled by rapid advances in portable, light-weight, and mobile EEG-imaging tools that could facilitate EEG/ERP applications within powerful developmentally-informative research designs [22,100].

## Supporting information

S1 Table Mean number of trials (SD) per stimulus type and group. (DOCX)

S2 Table Statistical comparisons on ADHD measures adjusted for genetic-relatedness and age (but not for IQ). (DOCX)

S3 Table Summary statistics and mean comparisons for performance scores and ERPs on the flanked CPT controlling for age and genetic-relatedness (and not for IQ). (DOCX)

S4 Table MZ and DZ cross-twin within-trait correlations for all ERPs and cross-twin cross-trait correlations between ADHD and all ERPs (from the flanked CPT) without regressing out IQ. (DOCX)

S5 Table Standardized estimates of genetic, shared and nonshared environmental contributions to the variance of ERPs (from the flanked CPT) using univariate twin analyses without regressing out IQ. (DOCX)

S1 Data NEAAT_CPT_ERP_data_ALZ_OS.sav. (SAV)
