# Supplementary material for: No evidence of associations between ADHD and event-related brain potentials from a continuous performance task in a population-based sample of adolescent twins
Source: PLoS One. 2019 Oct 4;14(10):e0223460. doi: 10.1371/journal.pone.0223460 (PMC6777760; doi:10.1371/journal.pone.0223460)
Supplement: S1 Table — (DOCX) [file pone.0223460.s001.docx]

| **S1 Table. Mean Number of Trials (SD) per Stimulus Type and Group** | | |
| --- | --- | --- |
|  | **ADHD** | **Control** |
| **Cue trials** | 57.20 (9.76) | 60.76 (8.54) |
| **Go trials** | 30.04 (5.39) | 31.65 (5.49) |
| **Nogo trials** | 27.04 (4.65) | 29.01 (4.27) |
| ADHD = Attention deficit/hyperactivity disorder | | |
