# Supplementary material for: No evidence of associations between ADHD and event-related brain potentials from a continuous performance task in a population-based sample of adolescent twins
Source: PLoS One. 2019 Oct 4;14(10):e0223460. doi: 10.1371/journal.pone.0223460 (PMC6777760; doi:10.1371/journal.pone.0223460)
Supplement: S2 Table — (DOCX) [file pone.0223460.s002.docx]

| **S2 Table. Statistical Comparisons on ADHD Measures Adjusted for Genetic-relatedness and Age (but not for IQ)** | | | |
| --- | --- | --- | --- |
|  | ***t*** | ***p*** |  |
| **Parents Conners Inattention subscale ^a^** | 8.93 | <.001 |  |
| **Parents Conners Hyperactivity-Impulsivity subscale ^a^** | 8.62 | <.001 |  |
| **Teachers Conners Inattention subscale ^b^** | 2.46 | .018 |  |
| **Teachers Conners Hyperactivity-Impulsivity subscale ^b^** | 1.59 | .120 |  |
| ADHD = Attention deficit/hyperactivity disorder  ^a^ Long version of the Parent Conners’ Rating Scale T-scores (Conners et al., 1998b) collected on the day of testing  ^b^ Long version of the Teacher Conners’ Rating Scale T-Scores (Conners et al., 1998a) collected by contacting teachers after completion of the testing session. | | | |
