# Supplementary material for: No evidence of associations between ADHD and event-related brain potentials from a continuous performance task in a population-based sample of adolescent twins
Source: PLoS One. 2019 Oct 4;14(10):e0223460. doi: 10.1371/journal.pone.0223460 (PMC6777760; doi:10.1371/journal.pone.0223460)
Supplement: S3 Table — (DOCX) [file pone.0223460.s003.docx]

| **S3 Table.** **Summary Statistics and Mean Comparisons for Performance Scores and ERPs on the Flanked CPT Controlling for Age and Genetic-relatedness (and not for IQ)** | | | | | |
| --- | --- | --- | --- | --- | --- |
|  | | **ADHD** | **Control** | ***t*** | ***p*** |
| **Performance** | | **(*n* = 52)** | **(*n* = 82)** |  |  |
| **Omissions** | | 1.94 (2.35) | 0.91 (1.60) | 1.81 | .075 |
| **Commissions** | | 2.56 (3.14) | 2.10 (2.43) | 0.80 | .425 |
| **MRT (ms)** | | 422.48 (66.24) | 396.45 (56.42) | 1.60 | .114 |
| **SDRT (ms)** | | 112.75 (53.03) | 86.84 (38.14) | 2.25 | .028 |
| **CV** | | 0.26 (0.11) | 0.21 (0.08) | 2.08 | .041 |
| **ERPs^a^** | | **(*n* = 50)** | **(*n* = 81)** |  | |
| **Cue-P3** | **Peak amplitude (μV)** | 11.93 (4.78) | 11.58 (4.96) | 0.29 | .770 |
|  | **Peak latency (ms)** | 545.49 (79.35) | 524.47 (72.90) | 0.64 | .526 |
| **Go-P3** | **Peak amplitude (μV)** | 16.48 (4.38) | 17.09 (6.24) | 1.18 | .240 |
|  | **Peak latency (ms)** | 422.85 (105.30) | 411.65 (97.89) | 0.67 | .505 |
| **NoGo-P3** | **Peak amplitude (μV)** | 13.40 (5.83) | 14.30 (5.74) | 0.81 | .420 |
|  | **Peak latency (ms)** | 432.43 (63.76) | 416.22 (67.07) | 0.53 | .596 |
| **Go-N2** | **Peak amplitude (μV)** | -5.39 (3.27) | -4.87 (4.25) | 0.54 | .593 |
|  | **Peak latency (ms)** | 312.45 (41.04) | 304.29 (41.92) | 0.57 | .570 |
| **NoGo-N2** | **Peak amplitude (μV)** | -7.14 (3.97) | -6.94 (4.49) | 0.17 | .869 |
|  | **Peak latency (ms)** | 297.67 (35.94) | 294.80 (31.61) | 0.06 | .950 |
| **CNV** | **Area amplitude (μV)** | 1.81 (1.06) | 1.71 (0.89) | 0.40 | .688 |
| CPT = continuous performance task; ADHD = attention deficit/hyperactivity disorder; MRT = mean reaction time; SDRT = standard deviation of reaction time; CV = coefficient of variation (SDRT/MRT); ERP = event-related potential  ^a^ ERPs were obtained from fewer participants than performance scores due to data loss in EEG pre-processing. | | | | | |
