# Supplementary material for: No evidence of associations between ADHD and event-related brain potentials from a continuous performance task in a population-based sample of adolescent twins
Source: PLoS One. 2019 Oct 4;14(10):e0223460. doi: 10.1371/journal.pone.0223460 (PMC6777760; doi:10.1371/journal.pone.0223460)
Supplement: S4 Table — (DOCX) [file pone.0223460.s004.docx]

| **S4 Table. MZ and DZ Cross-Twin Within-Trait Correlations for all ERPs and Cross-Twin Cross-Trait Correlations between ADHD and all ERPs (from the Flanked CPT) without Regressing Out IQ** | | | | | | |
| --- | --- | --- | --- | --- | --- | --- |
|  | | **Estimate (95% CI)^a^** | | | | |
|  |  | **Phenotypic Correlation with ADHD Grouping** | **Cross-Twin Within-Trait Correlation** | | **Cross-Twin Cross-Trait Correlation with ADHD** | |
|  |  |  | **MZ** | **DZ** | **MZ** | **DZ** |
| **Cue-P3** | **Amplitude** | -.05 [-.25 to .15] | **.45 [.17 to .65]*** | -.10 [-.45 to .29] | -.03 [-.31 to .25] | .08 [-.17 to 31] |
|  | **Latency** | .01 [-.17 to .19] | .12 [-.20 to .41] | .30 [-.11 to .58] | .28 [-.02 to .49] | -.05 [-.29 to .19] |
| **Go-P3** | **Amplitude** | -.07 [-.32 to .16] | .11 [-.21 to .40] | .18 [-.19 to .48] | -.23 [-.51 to .26] | .07 [-.19 to .32] |
|  | **Latency** | -.01 [-.20 to .18] | **.54 [.28 to .72]*** | -.05 [-.40 to .32] | .08 [-.17 to .32] | .19 [-.05 to .40] |
| **NoGo-P3** | **Amplitude** | -.12 [-.31 to .08] | **.43 [.08 to .66]*** | -.04 [-.34 to .27] | -.04 [.-.34 to .27] | .03 [-.21 to .26] |
|  | **Latency** | .07 [-.13 to .27] | .28 [-.03 to .53] | -.26 [-.56 to .13] | .07 [-.23 to .34] | -.02 [-.25 to .21] |
| **Go-N2** | **Amplitude** | -.09 [-.29 to .11] | .28 [-.04 to .54] | -.20 [-.50 to .17] | .08 [-.18 to .31] | .04 [-.21 to .28] |
|  | **Latency** | .14 [-.09 to .31] | .02 [-.26 to .29] | .13 [-.33 to .51] | -.23 [-.41 to .09] | .03 [-.23 to .28] |
| **NoGo-N2** | **Amplitude** | .00 [-.19 to .20] | **.45 [.18 to .65]*** | .24 [-.19 to .56] | -.12 [-.35 to .13] | .01 [-.24 to .26] |
|  | **Latency** | .04 [-.17 to .25] | .03 [-.46 to .49] | -.17 [-.42 to .10] | .03 [-.30 to .35] | -.19 [-.40 to .04] |
| **CNV** | **Amplitude** | .02 [-.17 to .20] | **.37 [.04 to .61]*** | .06 [-.27 to .38] | -.01 [-.25 to .24] | .10 [-.14 to .33] |
| MZ = monozygotic; DZ = dizygotic; ERP = event-related potential; ADHD = attention deficit/hyperactivity disorder; CPT = continuous performance task; CI = confidence intervals; CNV = continency negative variation  ^a^ The MZ and DZ correlations for ADHD were fixed to population values to account for the selected sample with rMZ = .76, rDZ = .38 and a threshold for population prevalence of 5%; all ERP outcomes were peak measures, except for CNV which was area amplitude.  ^*^ *p* < .05 | | | | | | |
