# Supplementary material for: No evidence of associations between ADHD and event-related brain potentials from a continuous performance task in a population-based sample of adolescent twins
Source: PLoS One. 2019 Oct 4;14(10):e0223460. doi: 10.1371/journal.pone.0223460 (PMC6777760; doi:10.1371/journal.pone.0223460)
Supplement: S5 Table — (DOCX) [file pone.0223460.s005.docx]

| **S5 Table. Standardized Estimates of Genetic, Shared and Nonshared Environmental Contributions to the Variance of ERPs (from the Flanked CPT) using Univariate Twin Analyses without Regressing out IQ** | | | |
| --- | --- | --- | --- |
|  | **Estimate (95% CI)^a^** | | |
|  | ***h^2^*** | ***c^2^*** | ***e^2^*** |
| **Cue-P3 amplitude** | .39 [ .00 to .61] | 0 [.00 to .34] | **.61 [.39 to .90]*** |
| **Go-P3 latency** | **.50 [.08 to .70]*** | 0 [.00 to .00] | **.50 [.30 to .78]*** |
| **NoGo-P3 amplitude** | .33 [.00 to .60] | 0 [.00 to .32] | **.67 [.40 to 1]*** |
| **NoGo-N2 amplitude** | .42 [.00 to .65] | .03 [.00 to .55] | **.55 [.35 to .81]*** |
| **CNV amplitude** | .34 [.00 to .58] | 0 [.00 to .42] | **.66 [.42 to .96]*** |
| ERP = event-related potential; CPT = continuous performance task; CI = confidence intervals; *h*^2^ = addictive genetic influences; *c^2^* = shared environmental influences; *e^2^* = nonshared environmental influences and measurement error  ^a^ For parsimony and simplicity, we focused on univariate models for selected ERP measures because 1) these showed significant MZ cross-twin within-trait correlation, 2) there were no significant phenotypic associations between ADHD grouping and any of the ERP measures, and 3) there were no significant cross-twin cross-trait correlations between any ERP measures and ADHD group  ^*^*p* < .05 | | | |
